# *Drosophila *insulin and target of rapamycin (TOR) pathways regulate GSK3 beta activity to control Myc stability and determine Myc expression in vivo

**DOI:** 10.1186/1741-7007-9-65

**Published:** 2011-09-27

**Authors:** Federica Parisi, Sara Riccardo, Margaret Daniel, Mahesh Saqcena, Nandini Kundu, Annalisa Pession, Daniela Grifoni, Hugo Stocker, Esteban Tabak, Paola Bellosta

**Affiliations:** 1 Department of Biology, City College of the City University of New York, New York, USA; 2 Department of Experimental Pathology, University of Bologna, Bologna, Italy; 3 Institute of Molecular Systems Biology, ETH, Zürich, Switzerland; 4 Courant Institute of Mathematical Science, New York University, New York, USA; 5 Department of Genetics and Development Columbia University, New York, New York, USA

## Abstract

**Background:**

Genetic studies in *Drosophila melanogaster *reveal an important role for Myc in controlling growth. Similar studies have also shown how components of the insulin and target of rapamycin (TOR) pathways are key regulators of growth. Despite a few suggestions that Myc transcriptional activity lies downstream of these pathways, a molecular mechanism linking these signaling pathways to Myc has not been clearly described. Using biochemical and genetic approaches we tried to identify novel mechanisms that control Myc activity upon activation of insulin and TOR signaling pathways.

**Results:**

Our biochemical studies show that insulin induces Myc protein accumulation in *Drosophila *S2 cells, which correlates with a decrease in the activity of glycogen synthase kinase 3-beta (GSK3*β *) a kinase that is responsible for Myc protein degradation. Induction of Myc by insulin is inhibited by the presence of the TOR inhibitor rapamycin, suggesting that insulin-induced Myc protein accumulation depends on the activation of TOR complex 1. Treatment with amino acids that directly activate the TOR pathway results in Myc protein accumulation, which also depends on the ability of S6K kinase to inhibit GSK3*β *activity. Myc upregulation by insulin and TOR pathways is a mechanism conserved in cells from the wing imaginal disc, where expression of Dp110 and Rheb also induces Myc protein accumulation, while inhibition of insulin and TOR pathways result in the opposite effect. Our functional analysis, aimed at quantifying the relative contribution of Myc to ommatidial growth downstream of insulin and TOR pathways, revealed that Myc activity is necessary to sustain the proliferation of cells from the ommatidia upon Dp110 expression, while its contribution downstream of TOR is significant to control the size of the ommatidia.

**Conclusions:**

Our study presents novel evidence that Myc activity acts downstream of insulin and TOR pathways to control growth in *Drosophila*. At the biochemical level we found that both these pathways converge at GSK3*β *to control Myc protein stability, while our genetic analysis shows that insulin and TOR pathways have different requirements for Myc activity during development of the eye, suggesting that Myc might be differentially induced by these pathways during growth or proliferation of cells that make up the ommatidia.

## Background

Genetic studies in *Drosophila *have identified Myc as well as components of insulin and target of rapamycin (TOR) signaling pathways as key regulators of growth [1,2]. Insulin and TOR signaling pathways are highly conserved. In *Drosophila*, binding of insulin-like peptides to the insulin receptor (InR) results in the activation and phosphorylation of *chico*, the ortholog of the insulin receptor substrates 1-4 (IRS1-4) [3]. This event leads to the production of phosphatidylinositol-3,4,5-triphosphate (PIP3) by phosphoinositide 3-kinase (PI3K), a reaction that is counteracted by the lipid phosphatase and tensin homolog (PTEN) [4,5]. PIP3 recruits several Ser/Thr kinases to the plasma membrane including Akt/PKB (Protein Kinase B)[6] and PDK1 (3'-phosphoinositide-dependent protein kinase-1) [7]. Activation of Akt results in the inhibition of glycogen synthase kinase 3-beta (GSK3β), a conserved kinase that not only controls energy metabolism by inactivation of glycogen synthase, but also regulates Wnt signaling by controlling βcatenin/*armadillo *[8] and Myc stability [9,10]. Activation of Akt also inhibits tuberous sclerosis complex 1 and 2 (TSC1/2), a binary-complex that negatively regulates Rheb, a GTPase upstream of TOR kinase responsible for activation of TOR complex 1 [11]. TOR is found in two complexes: TOR complex 1, which includes Raptor and LSt8 adaptor molecules, is sensitive to amino acids and is inhibited by rapamycin; and TOR complex 2, which is composed of LSt8 and Rictor adaptor molecules, and does not respond to amino acids or rapamycin [12,13]. Activation of TOR complex 1 results in phosphorylation of ribosomal protein kinase p-70-S6 (S6K) on threonine 398, and of eukaryotic translation initiation factor 4E-binding protein 1(4E-BP1), thereby triggering protein synthesis and initiation of translation [14,15]. Insulin and TOR activities are also balanced by a negative feedback mechanism that is activated when S6K is hyper-activated to counteract insulin activity. Under this condition, S6K phosphorylates IRS1-4/*chico *triggering its internalization and subsequent proteasomal degradation [16,17]. This feedback mechanism is reduced in pathological conditions, such as TSC syndromes where cells carrying mutations in *tsc1 *or *tsc2 *display an abnormal increase in size and exhibit constitutive phosphorylation of S6K [18]. In these cells, hyper-activation of S6K correlates with inactivation of GSK3β by phosphorylation of Serine 9, which results in c-Myc protein accumulation [18].

The *Drosophila dmyc *gene, called *diminutive *(*dm*), was identified as the sole ortholog of the human *c-myc *gene [19,20]. Analysis of *dm *target genes revealed a prominent role for Myc in the regulation of genes controlling ribosomal biogenesis and protein synthesis [21-24], thus animals carrying *dm *hypomorphic mutations are smaller due to a reduction in their protein synthesis and cell size [25-28]. This phenotype is reminiscent of hypomorphic mutants for *tor *or *s6k*, components of the TOR signaling pathway [29-31]. Signaling through the InR controls growth; mutants for the *Drosophila*-insulin-like peptides or of the adaptor molecule *chico/IRS *are also smaller because of fewer and smaller cells [3]. Recent genomic analysis in whole larvae showed a strong correlation between the targets of Myc and those of the TOR pathway; however, less overlap was found between the targets of Myc and those of PI3K signaling [23]. Whether Myc acts downstream or in parallel to PI3K is not totally clear and previous observations *in vivo*, in cells of the imaginal discs, indicate that activation of PI3K, even though it influenced growth and decreased the G1 phase of the cell cycle, did not significantly alter Myc protein levels [32]. However, in salivary glands, Myc expression was able to partially rescue the growth defect caused by lowering PI3K activity [27] suggesting that Myc activity might be acting downstream of PI3K signaling. Together, these data suggest that Myc could participate in the regulation of growth in response to insulin and TOR signaling, however a molecular link between Myc and these signaling pathways has not been clearly identified yet.

In this report, we provide evidence of a molecular mechanism for the Myc protein to be stabilized by insulin and amino acid signaling in *Drosophila *S2 cells, which converge to decrease the activity of GSK3β, a kinase responsible for Myc protein degradation. We found Myc protein regulated *in vivo *both by insulin and TOR pathways in epithelial cells of the imaginal discs. Using genetic analysis we demonstrate that Myc functions downstream of insulin and TOR to sustain proper growth of the eye during development.

## Results

### Insulin induces Myc protein accumulation in *Drosophila *S2 cells, with a mechanism that results in inactivation of GSK3β and is dependent on TOR complex 1 activity

**Figure 1:**
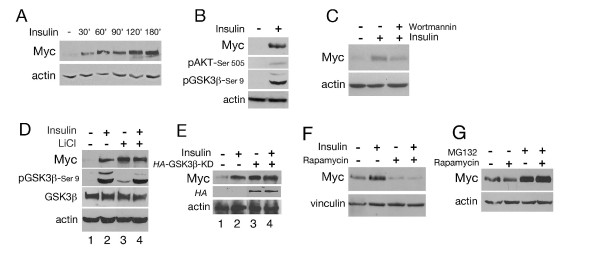
**Insulin induces Myc protein upregulation in *Drosophila *S2 cells, which depends on TOR signaling**. **(A) **Time course of Myc protein accumulation upon insulin treatment. Insulin was added to serum-starved S2 cells and Myc levels were analyzed by western blotting. **(B) **Insulin treatment increases Myc protein levels, correlating with phosphorylation of Akt on Ser 505 and of GSK3β on Ser 9. **(C) **Myc protein accumulation by insulin is inhibited in the presence of the PI3K inhibitor wortmannin. **(D and E)**. Blocking GSK3β activity with LiCl or using the GSK3β kinase dead mutant (GSK3β-KD), affects insulin-induced Myc protein accumulation. To block GSK3β activity, S2 cells were treated with LiCl (D), or transfected with a plasmid encoding for an HA-tagged GSK3β-KD that was previously demonstrated to reduce endogenous GSK3β kinase activity [10] (E). Treatment with insulin results in the accumulation of Myc protein (D and E, lane 2). Blocking GSK3β activity by LiCl (panel D, lane 3) or by the expression of GSK3β-KD (panel E, lane 3) enhanced Myc protein level, which was not further increased when insulin was added to cells where GSK3β activity was inhibited by LiCl or by the presence of its KD mutant (lane 4). Expression of the HA-GSK3β-KD was analyzed by western blotting using anti-HA antibodies. **(F) **Inhibition of TORC1 by rapamycin decreased insulin-induced Myc protein accumulation. Rapamycin, alone or together with insulin, was added for 2 h to serum-starved S2 cells; vinculin was used as a control for protein loading. **(G) **Blocking the proteosome pathway with MG132 inhibits Myc protein degradation by rapamycin. MG132 was added to the cells together with rapamycin. Myc protein levels were analyzed after 2 hours of treatment using anti-Myc antibodies; actin was used as a control for protein loading. GSK3β: glycogen synthase kinase 3-beta; LiCl: lithium chloride; KD: kinase dead; PI3K: phosphatidyl-inositol-3 kinase; TORC1: target of rapamycin complex 1.

Our previous observation that Myc protein stability in *Drosophila *S2 cells is reduced by GSK3β activity [10] led us to investigate if stimulation of insulin signaling, which inhibits GSK3β via Akt phosphorylation, could result in Myc protein accumulation. Treatment of *Drosophila *S2 cells with insulin induced an increase in Myc protein levels visible after 30 minutes of stimulation that was still detectable after 180 minutes of treatment (Figure 1A). This event was accompanied by a small increase in *dmyc-*RNA that peaked after 30 minutes and rapidly returned to baseline levels (Additional file 1). Myc protein accumulation by insulin was accompanied by phosphorylation of Akt on Ser 505, an event that correlated with phosphorylation of GSK3β on Ser 9 (Figure 1B), and was inhibited in the presence of the PI3K inhibitor wortmannin (Figure 1C). In order to analyze if GSK3β signaling contributes to insulin-induced Myc protein upregulation, S2 cells were treated with insulin in the presence of the GSK3β inhibitor lithium chloride (LiCl), or insulin was added to S2 cells expressing the GSK3β- kinase dead (KD) mutant, which was shown previously to reduce GSK3β activity [33].

In these experiments, addition of insulin increased Myc protein levels and this was accompanied by increased phosphorylation of GSK3β on Ser 9 (Figure 1D and 1E, lane 2). LiCl or expression of GSK3β-KD also increased endogenous Myc protein levels (Figure 1D and 1E, lane 3) as a result of inhibition of the endogenous GSK3β activity, which is known to control Myc protein stability [10]. Blocking GSK3β activity with LiCl or using GSK3β-KD, along with the addition of insulin, led to an increase in Myc, which was comparable to that of insulin alone (Figure 1D and 1E, compare lane 2 and 4).

Activation of insulin signaling enhances TOR activity by releasing the negative feedback of Akt on TSC1/2 [34-36]. We therefore treated S2 cells with rapamycin, an inhibitor of TOR complex 1 (TORC1), to analyze the contribution of TOR to insulin-mediated Myc upregulation. These experiments showed that rapamycin suppresses Myc protein accumulation by insulin (Figure 1F), and similar results were obtained for the regulation of *dmyc*-RNA (Additional file 2). These data also showed that rapamycin reduced endogenous Myc protein levels without affecting *dmyc*-RNA, suggesting that TOR signaling might regulate Myc protein stability. To better understand this mechanism we blocked the proteasome using MG132 and analyzed Myc protein level upon rapamycin treatment. These data showed that Myc protein degradation in the presence of rapamycin was completely suppressed by MG132 (Figure 1G) suggesting that TOR activity regulates Myc protein stability by mediating its degradation through the ubiquitin-proteosomal pathway.

### Amino acids and/or TOR signaling increases Myc protein stability by GSK3β inhibition in *Drosophila *S2 cells

**Figure 2:**
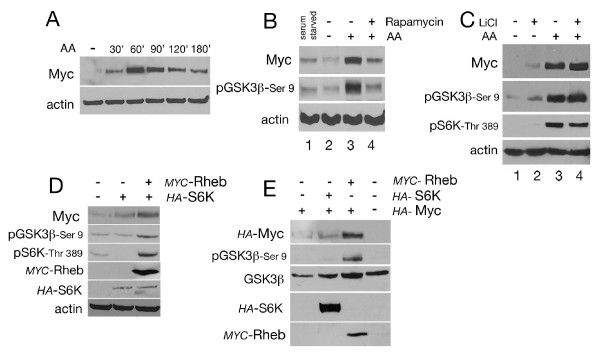
**Amino acid activation of TOR pathway decreases GSK3β activity and increases Myc protein levels**. **(A) **Time course of Myc protein accumulation upon AA treatment. Cells were serum-starved overnight and then bathed in AA-free medium for 30 min. AAs were added for the indicated time. Myc protein levels were analyzed by western blotting. **(B) **AA induced Myc protein accumulation through the activation of TORC1. S2 cells were serum-starved (lane 1) and bathed in AA-free medium for 30 min (lane 2). AAs were added back for 2 h in the absence (lane 3) or in the presence of rapamycin (lane 4). Myc accumulation corresponded to phosphorylation of GSK3β at Ser 9. **(C) **Inhibition of GSK3β by LiCl limits the ability of AAs to induce Myc protein accumulation. Cells were serum-starved and then bathed in AA-free medium. A solution of LiCl or AA, alone or together was added to the AA-free medium for 60 min. Accumulation of Myc protein correlates with phosphorylation of S6K on Thr 389 and of GSK3β on Ser 9. Of note: the effect of LiCl on Myc accumulation is less evident when cells are bathed in medium lacking AA than that observed when LiCl is added to cells bathed in complete Schneider medium and low serum (compare Myc levels in Figure 2C, lane 2 to that in Figure 1D lane 3). This difference could be explained by the possibility that cells bathed in AA-free medium have a reduced rate of protein synthesis. This could result in a delay of the mechanism that controls ubiquitin-induced Myc protein stability thus accounting for the reduced level of Myc observed when LiCl is added to AA-free medium. **(D) **Ectopic expression of Rheb and S6K increases Myc protein and correlates with phosphorylation of S6K on Thr 398 and of GSK3β on Ser 9. S2 cells were transfected with plasmids encoding for *HA-*S6K and *MYC-*Rheb. **(E) **S2-*tub*-Gal4 cells expressing *tubulin-*Gal4 were transfected to express *UAS*-*HA-*Myc, and co-transfected with *HA-*S6K and *MYC-*Rheb. Expression of the relative proteins was analyzed by western blotting using the indicated antibodies; actin and GSK3β were used as control loading. AA: amino acid; GSK3β: glycogen synthase kinase 3-beta; LiCl: lithium chloride; Rheb: Ras homolog enriched in brain; S6K: p70-S6 ribosomal protein kinase; TOR: target of rapamycin.

TOR signaling is activated by insulin and also by amino acids (AAs) [35,36]. To determine whether AAs directly controlled Myc protein accumulation, we serum-starved S2 cells and then performed a complete amino acid starvation by subsequently bathing them in an AA-free medium without serum. After 30 minutes, AAs were added back to the cells for the indicated times and Myc protein levels were analyzed by western blotting. As shown in Figure 2A, treatment with AAs increased Myc protein levels, which peaked between 60 and 90 minutes after treatment. Quantitative RT-PCR analysis showed that *dmyc*-mRNA was not significantly affected (Figure 1B in Additional file 1), suggesting that TOR signaling regulates Myc protein mainly at its post-translational level. AA starvation resulted in a reduction of Myc protein levels (Figure 2B, compare lane 1 and lane 2), which was increased by adding AAs back to the medium (Figure 2B, lane 3). This correlated with an increase in GSK3β phosphorylation of Ser 9. Myc upregulation by AAs was significantly reduced in the presence of rapamycin (Figure 2B, lane 4). In order to analyze if GSK3β activity contributes to TOR-induced Myc protein upregulation, we stimulated S2 cells with AAs in the presence of LiCl and analyzed whether AAs could still induce Myc protein accumulation in this condition.

These experiments showed that addition of LiCl to an AA-free medium slightly increased Myc protein level (Figure 2C, lane 2) but to a lesser extent than in medium with amino acids (compare with Figure 1D and 1E, lane 3). Addition of AAs increased Myc protein levels and was accompanied by an increase in phosphorylation of S6K on Thr 398 that correlated with phosphorylation of GSK3β on Ser 9 (Figure 2C, lane 3). Addition of LiCl together with AAs did not further increase Myc protein levels, an outcome similar to that observed with AA treatment alone (Figure 2C, compare lane 3 and 4). Similar results were obtained for the phosphorylation of S6K on Thr 398 or GSK3β on Ser 9.

We then analyzed if ectopic expression of Rheb and S6K resulted in accumulation of Myc protein. In these experiments the epitope-tagged form of Rheb (*MYC*-Rheb) and of S6K (*HA-*S6K) [37] were expressed in S2 cells and endogenous Myc protein was analyzed by western blot. These experiments showed that while S6K alone had a very small effect on Myc protein levels (Figure 2D), co-expression of S6K with Rheb was able to substantially induce Myc protein accumulation, an event that correlated with phosphorylation of S6K on Thr 398 and GSK3β on Ser 9. In order to understand if accumulation of Myc protein induced by Rheb and S6K was regulated at the post-transcriptional level, we co-expressed Rheb and S6K together with an epitope HA-tagged form of Myc [10]. In these experiments the UAS/Gal4 system [38] was used to express *UAS-HA-Myc *in a stable S2-line that constitutively expressed Gal4 under the *tubulin *promoter (S2-*tub *> Gal4). Rheb and S6K were co-expressed with HA-Myc and their protein levels were analyzed using anti-tag antibodies. These experiments showed that expression of S6K alone was not sufficient to induce significant changes in Myc protein levels, however expression of Rheb alone resulted in the accumulation of HA-Myc protein and this effect was accompanied by phosphorylation of GSK3β on Ser 9 (Figure 2E).

Taken together, these data show that TOR signaling controls Myc protein stability at the post-transcriptional level, which correlates with inhibition of GSK3β by its phosphorylation on Ser 9.

### Modulation of insulin and TOR signaling regulates Myc protein levels in epithelial cells of the wing imaginal discs

**Figure 3:**
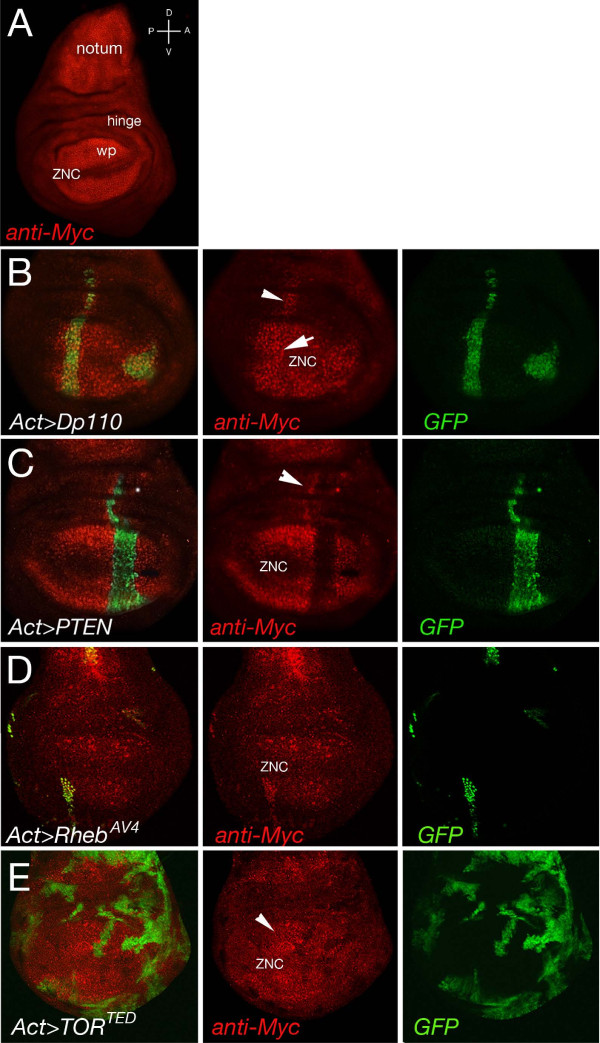
**Expression of components of the insulin and TOR signaling pathways modulates Myc protein levels in clones from the wing imaginal discs**. Analysis of Myc protein levels in flip-out clones expressing components of the insulin or TOR signaling pathways. **(A) **Endogenous Myc protein expression (red) in wing imaginal discs from third-instar larvae is higher in the notum and in the wing pouch (wp), while its levels is reduced in the hinge area and in the ZNC where Myc expression is inhibited by Wingless signaling. **(B-E) **Clones expressing the *actin > Gal4:PR *construct, together with *UAS-GFP*, were induced at 48 h AEL. Expression of the transgenes was induced using mifepristone and Myc protein levels analyzed by immunofluorescence after 5 h of treatment. **(B) **Clones expressing *UAS-Dp110 *showed Myc protein accumulation that was also visible in the ZNC where normally Myc expression is repressed (center, arrow). **(C) **Myc protein level was significantly reduced in clones expressing *UAS-PTEN*. **(D) **Clones expressing *UAS- UAS-Rheb^AV4 ^*showed increased Myc protein that was decreased in *UAS-TOR^TED ^*clones **(E)**. Notably, Myc protein was visibly induced non-autonomously in cells outside the border of the clones (arrowhead). AEL: after egg laying; TOR: target of rapamycin; wp: wing pouch; ZNC: zone of non-proliferative cells.

Our next step was to analyze if components of the insulin and TOR signaling pathways regulate Myc protein levels *in vivo *in cells from the wing imaginal discs. Because it was previously shown that reduction of components of the insulin and of TOR pathways *in vivo *resulted in clones of reduced size [3,31,39], we decided to take advantage of a conditional gene expression technique where the *Act > CD2 > PR*-*Gal4 *construct was used to temporally induce the expression of *UAS-*transgenes under the *actin *promoter through the addition of the synthetic steroid mifepristone [40]. Under these conditions, we generated clones expressing *UAS-Dp110 *or *UAS-PTEN *to modulate insulin signaling, or we activated the TOR pathway by expressing *rheb *using the *UAS-Rheb^AV4 ^*line, derived from insertion of a P element in the *rheb *locus [41]. The Myc protein level was analyzed in clones of cells from wing imaginal discs of third instar larvae after five hours of induction. Myc protein at this stage of development (Figure 3A, in red) is predominantly expressed within the cells of the presumptive notum and in the wing pouch, with the exception of the area of the hinge and a stripe of cells in the zone of non-proliferative cells (ZNC) located along the dorsal-ventral boundary, where Myc is transcriptionally repressed by Wingless activity [26,42,43]. These experiments showed that clones expressing Dp110, marked by co-expression of GFP, accumulate Myc protein (Figure 3B, red), which was also visible in cells from a clone that was generated within the ZNC, where normally Myc expression is repressed (arrow). In contrast, Myc protein was visibly reduced in clones overexpressing PTEN, a negative regulator of the insulin pathway (Figure 3C). Upregulation of TOR signaling, using *UAS-Rheb^AV^*, also induced the accumulation of Myc protein (Figure 3D); on the contrary Myc protein was reduced in clones expressing TOR*^TED^*, a mutant of TOR that functions as a dominant negative for TORC1 activity [44] (Figure 3E). Interestingly, these experiments revealed a strong non-autonomous accumulation of Myc protein in cells neighboring the clones, particularly visible in Dp110 and PTEN clones and to a lesser extent in Rheb*^AV4 ^*and TOR*^TED ^*clones (arrowhead). This was more evident when clones where positioned along the dorsal-ventral axis of the wing disc, suggesting that differences in Myc expression in cells at the border of the dorsal-ventral axis may induce changes that resulted in the regulation of Myc levels non-autonomously.

### Genetic interaction of Myc with members of the insulin and TOR signaling pathways in the adult eye

**Figure 4:**
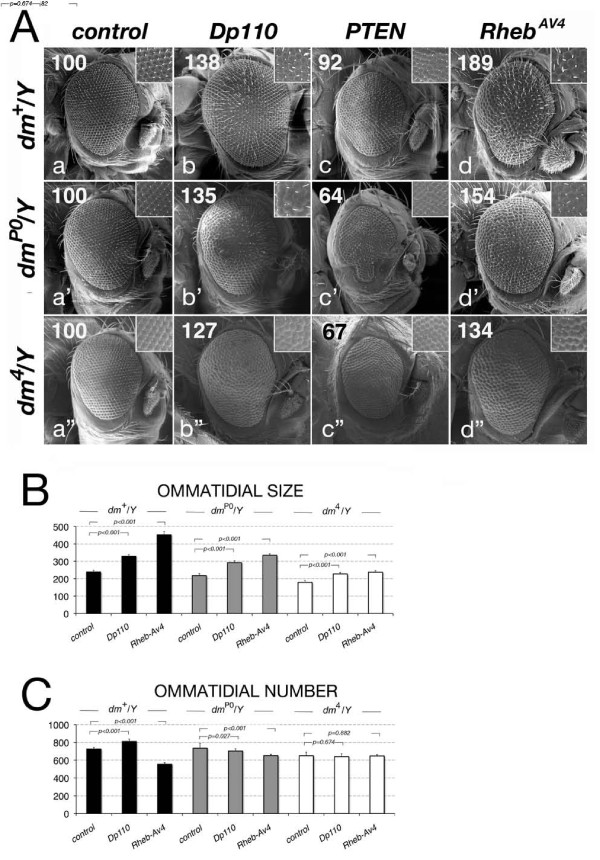
**Genetic interaction between Myc and components of the insulin and TOR pathways**. **(A) **Scanning electron micrographs of adult eyes from *ey- dm^+^/Y (a-d), ey- dm^P0^/Y (a'-d') *and *ey- dm^4^/Y (a"-d") *animals expressing the following transgenes: (a) control *yw*, (b) *UAS-Dp110*, (c) *UAS-PTEN*, and (d) *UAS-Rheb^AV4^*. All pictures are shown at the same magnification; posterior is to the left. Insets show higher-magnification views of the ommatidia. Numbers at the top right indicate cell size variation as compared to control (100) for each genotype also reported in panel B and C and Table 1. Histograms are representing the means of the **(B) **ommatidial size or **(C) **number of the indicated insulin and TOR components in different *dm *genetic backgrounds. The standard deviations were calculated based on the total number of animals, as indicated in Table 1. Note that the reduction of *dm *levels resulted in a small but significant decrease in ommatidial size in *ey-dm^P0^*/Y flies as compared to control *ey-dm^+^*/Y (Panel B and C, and Table S2 in Additional file 5; *P *< 0.001, calculated using Student *t *test). This difference was more pronounced in the eye of *dm^4^*/Y flies where the size of the ommatidia was reduced by 24% as compared to *ey-dm^+^*/Y flies. In addition, while the total number of the ommatidia was similar for *ey-dm^P0^*/Y flies and control *ey-dm^+^*/Y animals, flies with a null genetic background for *dm *(*ey-dm^4^/Y*) showed an 11% of reduction in the total number of the ommatidia as compared to *ey-dm^+^/Y *eyes [57]. TOR: target of rapamycin.

Given our biochemical and cellular evidence indicating that both the insulin and TOR pathways regulate Myc protein levels, we used a genetic approach to analyze the contribution of Myc to the growth exerted by these pathways. Using the UAS/Gal4 system in combination with flip-out techniques [45,46], we expressed components of the insulin or TOR pathways in the eye and analyzed their relative effect on growth by comparing the size and number of ommatidia from animals with different *dm *genetic backgrounds. In these experiments we recombined the *tubulin*-FRT-*dmyc*-FRT-Gal4 line, which we previously used to rescue viability of *dm^P0 ^*and lethality of *dm^4 ^*mutants, with the *eyeless*-Flp transgene [47]. This line contains the *dmyc-cDNA *located in a removable cassette between the *tubulin *promoter and Gal4. Upon FLP-mediated excision of *dmyc-cDNA*, the *tubulin *promoter drives Gal4 expression allowing for the expression of the UAS transgenes in the *eyeless *compartment (eye and antenna). This chromosome (hereafter called *ey*) was recombined in *wild-type *animals (*ey-dm^+^*) and in flies carrying the hypomorphic *dm^P0 ^*or the null *dm^4 ^*alleles (hereafter called *ey-dm^P0^*or *ey-dm^4^*). The size and number of the ommatidia in the eyes from control *wild-type *(*ey-dm^+^*) was compared to that from *ey-dm^P0 ^*or *ey-dm^4 ^*mutant animals expressing the different transgenes. The statistical significance of these data, across the different *dm *genetic backgrounds was calculated using the two-tailed *z *test (Appendix 1 in Additional file 3). Our first approach was to analyze if Myc activity modulates ommatidial size changes induced by components of the insulin pathway. This analysis revealed that even though expression of *UAS-Dp110 *increased the size of the ommatidia by 38% in a *wild-type dm^+ ^*background (Figure 4 and Table 1; *P *< 0.001, using a Student's *t *test) this effect was not dependent on Myc expression (Table S1 in Additional file 4; *P *= 0.4292, using a two-tailed *z *test) and only in *dm^4^/Y *null animals did Dp110 exhibit a weak but significant inhibition of its effect in increasing the size of the ommatidia (Table S1 in Additional file 4; *P *= 0.0367, using a two-tailed *z *test). By contrast, the increase in the total number of the ommatidia induced by Dp110 in *ey-dm^+^/Y *animals (Table 1; *P *< 0.001, *t *test) was significantly reduced in *dm^P0^*/Y and *dm^4^*/Y flies (Table S1 in Additional file 4; *P *< 0.001, two-tailed *z *test). Reduction of insulin signaling by PTEN showed a significant decrease in the size of the ommatidia in *ey-dm^+^*/Y animals (92%) (Figure 4, Table 1, *P *< 0.001, *t *test), and this effect was more pronounced in *ey-dm^P0^/Y *and *ey-dm^4^/Y *animals, where the size of the ommatidia was reduced to 64% and 67%, respectively (Table 1; *P *< 0.001, *t *test). PTEN also significantly reduced the total number of the ommatidia in the eyes of *ey-dm*^+ ^/Y and *dm^P0^*/Y flies (Table 1; *P *< 0.001, *t *test). Statistical analysis of relative growth across the different *dm *genetic backgrounds showed that this reduction was highly significant for both *dm *backgrounds (Table S1 in Additional file 4; *P *< 0.001, two-tailed *z *test). These data suggest that Myc activity significantly contributes to insulin signaling-induced growth in the eye.

**Table 1:** Genetic interaction between Myc and components of insulin and target of rapamycin signaling.

Genotype	Total number ommatidia	Ommatidium cell size (μm^2^)	% number^a^	% cell size^b^
*ey- dm*^+ ^*/Y; +/+; +/+ *	726 ± 17 (15)	239 ± 14 (15)	100	100

*ey- dm*^+ ^*/Y; UAS-Dp110/+*	811 ± 31 (06) *	329 ± 10 (06) *	112	138

*ey- dm*^+ ^*/Y; UAS-PTEN/+*	538 ± 25 (13) *	220 ± 11 (09) *	074	092

*ey- dm*^+ ^*/Y; UAS-S6K/+*	692 ± 37 (10) **	251 ± 12 (11) **	095	105

*ey- dm*^+ ^*/Y; UAS- Rheb^-AV4 ^/+*	555 ± 18 (06) *	452 ± 19 (06) *	076	189

*ey- dm*^+ ^*/Y; UAS-TOR-^TED ^/+*	579 ± 55 (11) *	214 ± 13 (11) *	080	089

*ey- dm*^P0^*/Y; +/+; +/+*	735 ± 16 (12)	218 ± 09 (12)	100	100

*ey- dm *^P0^*/Y; UAS-Dp110/+*	703 ± 41 (06) **	293 ± 12 (06) *	096	135

*ey- dm *^P0 ^*/Y; UAS-PTEN/+*	450 ± 16 (06) *	140 ± 09 (06) *	061	064

*ey- dm *^P0 ^*/Y; UAS-S6K/+*	684 ± 35 (09) *	216 ± 15 (13)	093	099

*ey- dm*^P0 ^*/Y; UAS- Rheb^-AV4 ^/+*	651 ± 31 (07) *	335 ± 09 (09) *	088	154

*ey- dm *^P0^*/Y; UAS-TOR-^TED ^/+*	n.d.	n.d.	n.d.	n.d.

*ey- dm*^4^*/Y; +/+; +/+*	650 ± 44 (17)	179 ± 11 (10)	100	100

*ey- dm*^4^*/Y; UAS-Dp110/+*	641 ± 66 (11)	228 ± 08 (06) *	100	127

*ey- dm*^4^*/Y; UAS-PTEN/+*	458 ± 42 (06) *	120 ± 13 (06) *	070	067

*ey- dm*^4^*/Y; UAS- Rheb-^AV4 ^/+*	647 ± 29 (12)	238 ± 22 (08) *	100	132

*ey- dm*^4 ^*/Y; UAS-TOR-^TED ^/+*	n.d.	n.d.	n.d.	n.d.

Standard deviations (±) are calculated based on the total number of the animals (reported in parenthesis); ^a^Percentage of increase in size compared to relative control (100); ^b ^Percentage increase in cell number compared to relative control (100); *p-values *calculated using a Student *t *test: * *P *< 0.001; ** *P *< 0.05; n.d. = not determined. Of note: overexpression of PTEN in *ey-dm^P0^/Y *animals resulted in a misshaped eye phenotype (Figure 4A, Panel c') with a 98% penetrance, which might have led to an underestimation of the total number of ommatidia and therefore affected our statistical analysis.

Genetic analysis using components of TOR signaling showed that, while expression of p70-S6K moderately affected the size of the ommatidia (Table 1, *P *< 0.05, *t *test), expression of the *UAS-Rheb^AV4 ^*allele [39] showed an 89% increase of ommatidia size in *ey-dm*^+ ^/Y flies (Table 1, *P *< 0.001, *t *test) and this effect was drastically reduced in *ey-dm^P0^/Y *and *ey-dm^4^/Y *animals (Table 1, *P *< 0.001, *t *test). This event was statistically significant as demonstrated by our analysis of the relative size increases across the different *dm *genetic backgrounds (Table S1 in Additional file 4; *P *< 0.001, *z *test).

In summary these data suggests that Myc functions downstream of TOR signaling to control the growth of the size of the ommatidia.

While performing these experiments we noticed that expression of components of TOR signaling, and in particular of the strong *Rheb^AV4 ^*allele, had a significant negative effect on the total number of ommatidia (Table 1; *P *< 0.001, *t *test). Moreover, this effect was rescued by reducing *dmyc *levels (Figure 4, Table 1). To understand the molecular mechanisms that caused Rheb to reduce the ommatidia number, imaginal discs from third instar larvae expressing *UAS- Rheb^AV4 ^*transgenes, were examined for defects in cell proliferation or for increased cell death. Imaginal eye discs from *ey-dm^P0^/Y *or wild-type *ey-dm*^+^/Y animals carrying the *UAS-Rheb^AV4 ^*transgene were subjected to bromodeoxyuridine (BrdU) labeling to detect DNA replication (S phase), or immunostained with anti-active caspase-3 to detect apoptotic cells. This analysis revealed that, while no significant changes were observed in the pattern of BrdU labeling between the different genotypes (Additional file 6), a significant increase in the number of caspase-3 positive cells in the antennal and eye imaginal discs of *ey-dm*^+^/Y; *UAS-Rheb^AV4 ^*/+ larvae was seen, which was significantly reduced in *ey-dm*^P0^/Y; *UAS-Rheb^AV4 ^*/+ animals (Additional file 7). This highlights a potential mechanism for TOR signaling to induce cell death when growth is in excess.

## Discussion

Previous studies in vertebrates have indicated a critical function for Myc downstream of growth factor signaling including insulin-like growth factor, insulin and TOR pathways [18,48-50]. In *Drosophila*, despite a few notes that Myc transcriptional activity acts downstream of insulin and TOR pathways [23,24], no clear molecular mechanisms linking these pathways to Myc have been elucidated yet.

**Figure 5:**
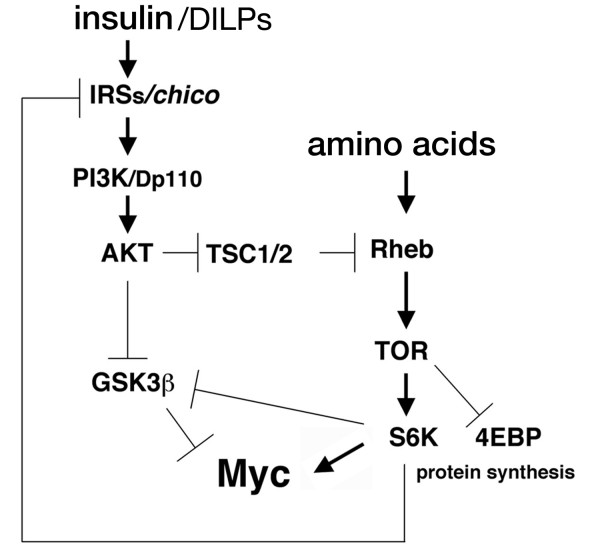
**Model showing the proposed relationship between Myc and the insulin and TOR signaling pathways**. AA: amino acids; DILPs: *Drosophila *insulin-like peptides; IRS: insulin-receptor substrate; PI3K: phosphatidylinositol-3 kinase; Rheb: Ras homolog enriched in brain; S6K: p-70-S6 ribosomal protein kinase; TSC: tuberous sclerosis complex; TOR: target of rapamycin.

We previously demonstrated that inhibition of GSK3β prevents Myc degradation by the proteasome pathway [10]. In this report, we further unravel the pathways that control Myc protein stability and show that signaling by insulin and TOR induce Myc protein accumulation by regulating GSK3β activity in S2 cells. GSK3β is a constitutively active kinase that is regulated by multiple signals and controls numerous cellular processes [8]. With our biochemical data we propose that GSK3β acts as a common point where insulin and TOR signaling converge to regulate Myc protein stability (Figure 5). In particular, we showed that activation of insulin signaling induces activation of Akt, an event that is accompanied by GSK3β phosphorylation on Ser 9 that causes its inactivation and Myc protein to stabilize (Figure 1B). Interestingly, insulin-induced Myc protein accumulation, when GSK3β activity was blocked by the presence of LiCl or by expression of GSK3β-KD, was similar to that obtained with insulin alone. Since we showed that activation of insulin signaling leads to GSK3β inhibition and to an increase in Myc protein, if insulin and GSK3β signaling were acting independently, we would expect that activation of insulin signaling concomitantly with the inhibition of GSK3β activity would result in a higher level of Myc than that obtained with insulin or LiCl alone. Our results instead showed a similar level of Myc protein accumulation with insulin in the presence of GSK3β inhibitors as compared to insulin alone (Figure 1D and 1E, compare lane 2 and 4), supporting the hypothesis that GSK3β and insulin signaling, at least in our experimental condition, depend on each other in the mechanism that regulates Myc protein stability.

In a similar biochemical approach, we analyzed the effect of AAs on Myc protein stability and how TOR signaling is linked to mechanisms that inactivate GSK3β to stabilize Myc protein in S2 cells. In these experiments we were able to demonstrate that AAs increased Myc protein stability, and we also showed that treatment with rapamycin, an inhibitor of TORC1, reduced insulin-induced Myc upregulation. The reduction of Myc protein accumulation by rapamycin was blocked by inhibition of the proteasome pathway, linking TOR signaling to the pathway that controls Myc protein stability (Figure 1F). TORC1 is a central node for the regulation of anabolic and catabolic processes and contains the central enzyme Rheb-GTPase, which responds to amino acids by activating TOR kinase to induce phosphorylation of p70-S6K and 4E-BP1 [14,15]. Our analysis of the molecular mechanisms that act downstream of TOR to regulate Myc stability shows that AA treatment induces p70-S6K to phosphorylate GSK3β on Ser 9, an event that results in its inactivation and accumulation of Myc protein (Figure 2).

Reducing GSK3β activity with LiCl, in medium lacking AAs, resulted in a slight increase in Myc protein levels (Figure 2C, lane 1 and 2). Adding back AAs lead to a substantial increase in Myc protein levels, which did not further increase when AAs where added to cells in the presence of the GSK3β inhibitor LiCl (Figure 2C, lane 3 and 4). These events were accompanied by phosphorylation of S6K on Thr 398, which correlated with phosphorylation of GSK3β on Ser 9. From these experiments we conclude that TOR signaling also converges to inhibit GSK3β activity to regulate Myc protein stability (Figure 5). However, we need to point out that since AAs alone increased Myc protein levels to a higher extent than that observed with LiCl alone (Figure 2C, compare lane 2 and 3), our experiments also suggest that Myc protein stability by TOR signaling is not solely directed through the inhibition of GSK3β activity, but other events and/or pathways contribute to Myc regulation. In conclusion, our biochemical experiments demonstrate that GSK3β acts downstream of insulin and TOR pathways to control Myc stability, however we do not exclude that other pathways may control Myc protein stability upon insulin and amino acids stimulation in S2 cells.

Reduction of insulin and TOR signaling *in vivo *reduces cell size and proliferation, and clones mutant for *chico*, the *Drosophila *orthologue of IRS1-4, or for components of TOR signaling, are smaller due a reduction in size and the number of cells [29,31,39,51]. Our experiments showed that reducing insulin signaling by expression of PTEN or using TOR*^TED^*, a dominant negative form of TOR, decreased Myc protein levels in clones of epithelial cells of the wing imaginal discs, while the opposite was true when these signals were activated using Dp110 or *Rheb^AV4 ^*(Figure 3). Those experiments suggested that the mechanism of regulation of Myc protein by insulin and TOR pathways was conserved also *in vivo *in epithelial cells of the larval imaginal discs.

During these experiments we also noted that Myc protein was induced in the cells surrounding and bordering the clones (non-autonomously), particularly when clones where positioned along the dorsal-ventral axis of the wing disc. This upregulation of Myc protein was not restricted to components of the insulin signaling pathway since we also observed it in cells surrounding the clones mutant for components of the Hippo pathway [52] or for the tumor suppressor *lethal giant larvae (lgl)*, which upregulates Myc protein cell-autonomously [53]. We suspect that this non-autonomous regulation of Myc may be induced by a novel mechanism that controls proliferation of cells when 'growth' is unbalanced. We can speculate that clones with different growth rates, caused by different Myc levels, might secrete factors to induce Myc expression in neighboring cells. As a consequence, these Myc-expressing cells will speed up their growth rate in an attempt to maintain proliferation and tissue homeostasis. Further analysis is required to identify the mechanisms responsible for this effect.

In order to distinguish if Myc activity was required downstream of insulin and TOR signaling to induce growth, we performed a genetic analysis. The ability to induce growth and proliferation was measured in the eye by measuring the size and number of the ommatidia from animals expressing members of the insulin and TOR pathways in different *dm *genetic background (*dm^+^, dm^P0 ^*and *dm^4^*). Our data showed that Dp110 increased the size and number of the ommatidia, however only the alteration in the total number was dependent on *dm *levels. These data suggest that Myc is required downstream of insulin pathway to achieve the proper number of ommatidia. However, when insulin signaling was reduced by PTEN, a significant decrease in the size of ommatidia was seen and it was dependent on *dm *expression levels, suggesting that Myc activity is limiting for ommatidial size and number. Activation of TOR signaling induces growth [2], and our genetic analysis showed that Myc significantly contributes to the size of the ommatidial cells thus suggesting that Myc acts downstream of TOR pathway to control growth.

Recent genomic analysis showed a strong correlation between the targets of Myc and those of the TOR pathway [24], implying that they may share common targets. In support of this observation our mosaic analysis with a repressible cell marker (MARCM) experiments in the developing wing disc showed that overexpression of Myc partially rescues the growth disadvantage of clones mutant for the hypomorphic *Rheb^7A1 ^*allele (Additional file 8), further supporting the idea that Myc acts downstream of TOR to activate targets that control growth in these clones.

Our genetic interaction revealed a stronger dependence on Myc expression when Rheb was used as opposed to S6K (Table 1). A possible explanation for this difference could lie in the fact that S6K is not capable of auto-activation of its kinase domain unless stimulated by TOR kinase. TOR activity is dependent on its upstream activator Rheb; consequently the enzymatic activity of the Rheb/GTPase is the limiting factor that influences S6K phosphorylation and therefore capable of maximizing its activity [54].

Interestingly, these experiments also showed that activation of TOR signaling has a negative effect on the number of ommatidia, and this correlates with the ability of Rheb*^AV4 ^*to induce cell death during the development of the eye imaginal disc. Rheb-induced cell death was rescued in a *dm^P0 ^*mutant background, which led us to speculate that 'excessive' protein synthesis, triggered by overexpression of TOR signaling, could elicit a Myc-dependent stress response, which induces apoptosis. Alternatively, high protein synthesis could result in an enrichment of misfolded proteins [55] that may result in a stress response and induces cell death. Further analysis is required to delineate the mechanisms underlying this process.

## Conclusions

Our analyses provide novel genetic and biochemical evidences supporting a role for Myc in the integration of the insulin and TOR pathway during the control of growth, and highlights the role of GSK3β in this signaling. We found that insulin signaling inactivates GSK3β to control Myc protein stability, and a similar biochemical regulation is also shared by activation of the TOR pathways. In support of this data, a recent genomic analysis in whole larvae showed a strong correlation between the targets of Myc and those of the TOR pathway; however, less overlap was found between the targets of Myc and those of PI3K signaling [23].

Statistical analysis applied to our genetic interaction experiments revealed that, in the *Drosophila *eye, proliferation induced by activation of the insulin pathway is sensitive to variations in Myc levels, while a significant interaction was seen mostly when TOR increased cell size. Our data therefore suggests that there is a correlation between Myc and the InR signaling and it is expected that the InR pathway also shares some transcriptional targets with Myc. Indeed, we found an overlap between the targets induced by insulin and Myc in *Drosophila *S2 cells (PB, unpublished data) and these targets have also been reported in transcriptome analyses in the fat body upon nutritional stress [24], suggesting that Myc acts downstream of InR/PI3K and TOR signaling and that this interaction might be specific to some tissues or in a particular metabolic state of the cell.

## Methods

### Fly lines

Fly stocks were obtained from the Bloomington stock center including *w; UAS-Rheb^AV4^/TM6b which is *derived from *P-*element inserted in the *rheb *locus [41] , *UAS-Dp110 *[56], *UAS-PTEN *[5] , with the exception of *w; UAS-TOR^TED ^*a mutant that expresses the toxic extended domain of TOR protein (Thomas Neufeld, UMN, MN, USA), *UAS-HA-Myc *[47], *yw; UAS-Rheb and w; FRT82, Rheb^7A1^/TM6b *(Hugo Stocker, ETH-ZH, Switzerland), *w; hs-Flp; tub-Gal4, UAS-GFP; FRT82 (hs-CD2 y+)-tub-Gal80 (w+) *(Myriam Zecca, Columbia University, NY, USA); *UAS-GFP;Ay-Gal4:PR[3]/TM6b *(Kenneth Irvine, Rutgers, NJ, USA [40]), *ey*-*dm^4 ^*= *yw dm^4 ^tubulin*-FRT-*dmyc*-cDNA-FRT-Gal4 *ey-Flp/Y *[57], *ey*-*dm^P0 ^*= *yw dm^P0^tubulin*-FRT-*dmyc*-cDNA-FRT-*Gal4*, *ey-Flp/Y*, and *ey*-*dm^+ ^*= *yw dm^+ ^tubulin*-FRT-*dmyc*-cDNA-FRT-*Gal4, ey-Flp/Y*.

### Cell culture and western blot

*Drosophila *Schneider S2 cells or the line expressing the plasmid *tubulin*-Gal4 (S2*-tub-Gal4) *[10] were grown at 25°C using Schneider medium (Invitrogen, GIBCO, Carlsbad, CA, USA) supplemented with 10% heat-inactivated FCS and 100 I.U. of penicillin/streptomycin (Invitrogen, GIBCO, Carlsbad, CA, USA). Insulin and AA treatments: S2 cells were serum-starved in 0.5% serum for 12 h and then various chemical inhibitors were added to the medium with or without insulin (from porcine, Sigma, St Louis, MO, USA 1 μM final concentration). For AA treatment, S2 cells were serum-starved as described above and further incubated for 30 min in medium without amino acids, made from the recipe of *Drosophila *Schneider medium (Invitrogen, GIBCO, Carlsbad, CA, USA) containing 0.5% dialyzed FCS. A 2× solution containing the same concentration of AAs as the *Drosophila *Schneider medium was added to induce an AA response for the indicated times. Cells were washed in PBS and lysed in a buffer containing 50 mM HEPES, pH 7.4; 250 mM NaCl; 1 mM EDTA, 1% Triton and protease and phosphatases inhibitors (Roche, Mannheim, Germany). Protein concentration was measured using the Bio-Rad protein assay. Western blot analysis was performed using the following primary antibodies: anti-*Drosophila *Myc mAb [10]; anti-HA (Roche, Mannheim, Germany), anti-actin mAb and anti vinculin mAb (Sigma, St. Louis, MO, USA); anti-*Drosophila *phospho-Akt, anti-phospho-GSK3β anti-phospho-S6K and anti GSK3β (Cell Signaling Technology inc. Danvers, MA, USA). Chemical inhibitors were used at 1 μM for rapamycin, 100 nM for wortmannin, and 50 mM for LiCl. Plasmids encoding HA-S6K (pAct-*HA-s6k*) and Myc-Rheb (pAct-*Myc-Rheb*), were a gift from Duojia J. Pan [37]. Transfection of S2 cells or of S2-tub-Gal4 was performed using Cellfectin reagent (Invitrogen, Carlsbad, CA, USA). The efficiency of transfection in S2 cells was analyzed by co-transfecting *tub-Gal4 *with UAS-GFP plasmids, and was found in the range of 30% to 60% depending on the experiment. Experiments were repeated at least three times with similar results.

### Generation of inducible Flp-out clones

Flp-out clones were generated using *UAS-GFP;AyGal4:PR[3]/TM6b *flies [40] by heat shock for 20 min at 37°C. Heat shock was performed 48 h after egg laying (AEL). Gal4:PR was activated by transferring larvae to instant food (Instant *Drosophila *Medium, Connecticut Valley Biological, MA, USA) containing RU486 (mifepristone, Sigma, St. Louis, MO, USA). Two grams of instant food were mixed with 7 mL of water previously supplemented with RU486, resulting in a final concentration of 20 μg/ml of mifepristone. Immunostaining protocol is reported in the Additional file 9: Supplementary Material and Methods.

### Analysis of ommatidial size and number

Flies were reared at 25°C under reproducible growth conditions and age matched (three-day-old males) before determining the ommatidial size and number. Total ommatidial number was counted on scanning electron micrographs from animals of the indicate genotypes. Size of the ommatidia was calculated by measuring the area of 20 ommatidia located at the center of the eye using Adobe Photoshop 7.0, as described previously [47]. At least eight animals of each genotype were measured.

### Statistical analysis

To perform this statistical analysis we considered two components: an estimation of the relative size and number of the ommatidia and their variability, and a standard two-sided *z *test to establish the significance of the differences of our data within the different *dm *genetic backgrounds. Means comparison between groups of animals was made using a Student's *t *test. Data in Table 1 are presented as means. Standard deviations and the number of animals are represented in parenthesis. A two-tailed *z *test analysis was performed to calculate the *p *values for the hypothesis that the relative growth of the ommatidial size and number for the various phenotypes are equal (see Appendix 1 in Additional file 3 and Table S1 in Additional file 4).

## Abbreviations

AA: amino acid; AEL: after egg laying; BrdU: bromodeoxyuridine; EDTA: ethylenediaminetetraacetic acid; FCS: fetal calf serum; GFP: green fluorescent protein; GSK3β: glycogen synthase kinase 3-beta; GSK3β-KD: GSK3β kinase dead; InR: insulin receptor; IRS: insulin-receptor substrate; LiCl: lithium chloride; MARCM: mosaic analysis with a repressible cell marker; PBS: phosphate buffered saline; PIP3: phosphatidylinositol-3,4,5-triphosphate; PI3K: phosphatidyl-inositol-3 kinase; PTEN: phosphatase and tensin homolog; Rheb: Ras homolog enriched in brain; RT-PCR: reverse transcription polymerase chain reaction; S6K: p70-S6 ribosomal protein kinase; TOR: target of rapamycin; TORC1: TOR complex 1; TSC: tuberous sclerosis complex; ZNC: zone of non-proliferative cells; 4E-BP1: eukaryotic translation initiation factor 4E-binding protein 1.

## Authors' contributions

FP, SR, MD, MS and NK performed the experiments. AP, DG and HS participated in the discussion and conceiving part of the experiments. ET formulated the mathematics and statistics analysis. PB participated in the discussion and conceiving the experiments and wrote the manuscript. All authors read and approved the final manuscript.

## Supplementary Material

Additional file 1 **Quantitative RT-PCR comparing the transcript levels for *dm*, *cyclin D *and *cyclin E *in *Drosophila *S2 cells upon insulin (A) or amino acids (B) treatment**. Cells were treated with insulin or AAs and RNA was extracted at the indicated times. qRT-PCRs were performed to analyze expression of *diminutive (dm), cyclin D *and *cyclin E *RNAs. The sequences of the primers used are available in Additional file 8; Supplementary Material and Methods. *actin *was used as the internal control. Error bars indicate the standard deviations (±) calculated on the average of three separate experiments. Click here for file

Additional file 2 **Quantitative RT-PCR of *dm *and its target *fibrillarin *in *Drosophila *S2 cells upon insulin treatments and in the presence of rapamycin**. Cells were treated with insulin or rapamycin alone and together as indicated in the figure; *rp49 (ribosomal protein 49) *was used as internal control. Similar results were obtained using *actin *as a control (not shown). Error bars indicate the standard deviation (±) calculated from three independent experiments. Click here for file

Additional file 3 **Appendix 1**. Significance analysis of the conjectured growth for ommatidial number and size used in the experiments outlined in Figure 4 and Table 1. The *P-*values of a two-tailed *z *test for the analysis are represented in Table S1 in Additional file 4. Click here for file

Additional file 4 **Table S1. Representation of the relative increase of the size for each ommatidium and their total number**. The values represent the percentage of increase in size (a-Size) or number (a-Number) as compared to their control and relative genetic background (see also data in Table 1). da is the standard deviations and the total number of animals used is indicated in Table 1. To establish the significance of the relative differences of the data within the different *dm *genetic background, we calculate the *P-values *using a standard two-sided *z*-test (the formula used is represented in Appendix 1 of Additional file 3). * complete genotype: the construct *tubulin*-FRT-*dmyc*-cDNA-FRT-*Gal4, ey-Flp/Y *was recombined into the *dm^+^, dm^po^or dm^4 ^*genetic background. Click here for file

Additional file 5 **Table S2. Analysis of the ommatidial size and number in animals with different *dm *genetic background**. The total number of ommatidia and the relative size of each ommatidium are indicated. Standard deviations (±) are calculated based on the total number of the animals reported in parenthesis. Values represent the relative increase in the size (a) or number (b) of the ommatidia compared to the values in their genetic background (100). *P-values *are calculated from Student *t *test and are reported for the calculation of ommatidia number (c) and size (d). * complete genotype: the construct *tubulin*-FRT-*dmyc*-cDNA-FRT-*Gal4, ey-Flp/Y *was recombined into the *dm^+^, dm^po^or dm^4 ^*genetic background. Click here for file

Additional file 6 **BrdU-labeling in the eye imaginal discs from third instar larvae expressing *Rheb^AV4 ^*transgene in *wild-type dm^+^*/Y (A) or in hypomorphic *dm^P0^*/Y animals (B)**. Expression of the *UAS-Rheb^AV4 ^*did not significantly alter the S phase in the cells of the eye imaginal disc, visualized by BrdU labeling (red). Nuclei are labeled with DAPI (blue). Posterior is to the left. Click here for file

Additional file 7 **Expression of *Rheb^AV4 ^*induces Myc-dependent apoptosis**. Third instar eye imaginal discs from *ey-dm^+^/*Y or *ey-dm^P0^/*Y larvae carrying the *UAS-Rheb^AV4 ^*transgene **(A-B) **or control chromosome **(C-D) **were tested for the presence of apoptotic cells. Discs were stained with anti-active caspase 3 antibody (red) to visualize cell death, or with anti ELAV (green) to mark the differentiated neuronal cells posterior to the morphogenetic furrow (MF). DAPI staining (blue) indicates nuclei. **(E) **Quantification of caspase-positive cells in the region posterior to the MF of the indicated genotype (visible in insets). Error bars indicate standard deviation (±) calculated from six independent eye imaginal discs. *P *< 0.001for *t *test for *ey- dm^+^/*Y; *UAS-Rheb^AV4 ^*vs. *ey- dm^P0^/*Y; *UAS-Rheb^AV4 ^*while comparisons within the other genotypes resulted in *P *> 0.1. **(F) **Photo of an eye imaginal disc from third-instar *ey- dm^+^*/Y; *UAS-GFP *larvae highlighting the territory where the *eyeless*-Gal4; *UAS-GFP *transgene is expressed. Posterior is to the left. Click here for file

Additional file 8 **MARCM showing a partial rescue of *Rheb^7A1 ^*hypomorphic mutant clones by Myc overexpression**. *Rheb^7A1 ^*homozygous mutant clones suffer of growth disadvantage [39]. These clones, induced at 72 h AEL and marked by GFP expression, are significantly smaller than *wild-type *siblings, which are marked by CD2 staining (red). The growth defect of *Rheb^7A1 ^*mutant clones (**A) **is partially rescued by expression of Myc **(B)**. Those clones are visible by co-expression with GFP, while *wild-type *clones are marked by the expression of CD2 and visualized by immunofluorescence using anti-CD2 antibodies. To generate MARCM clones the line *hs-flp, tub-Gal4 (w+), UAS-GFP (w+); FRT82 [hsCD2 (y+)] tub-Gal80 *was crossed with the line *w; FRT82 Rheb^7E1^/TM6b ***(A) **or with *w; UAS-dMyc; FRT82 Rheb^7E1^/TM6b ***(B)**. (see Supplementary Material and Methods in Additional file 9). Click here for file

Additional file 9 **Supplementary Material and Methods**. Click here for file
