# Supplementary figures and images for: Drosophila insulin and target of rapamycin (TOR) pathways regulate GSK3 beta activity to control Myc stability and determine Myc expression in vivo
Source: BMC Biol. 2011 Sep 27;9:65. doi: 10.1186/1741-7007-9-65 (PMC3235970; doi:10.1186/1741-7007-9-65)

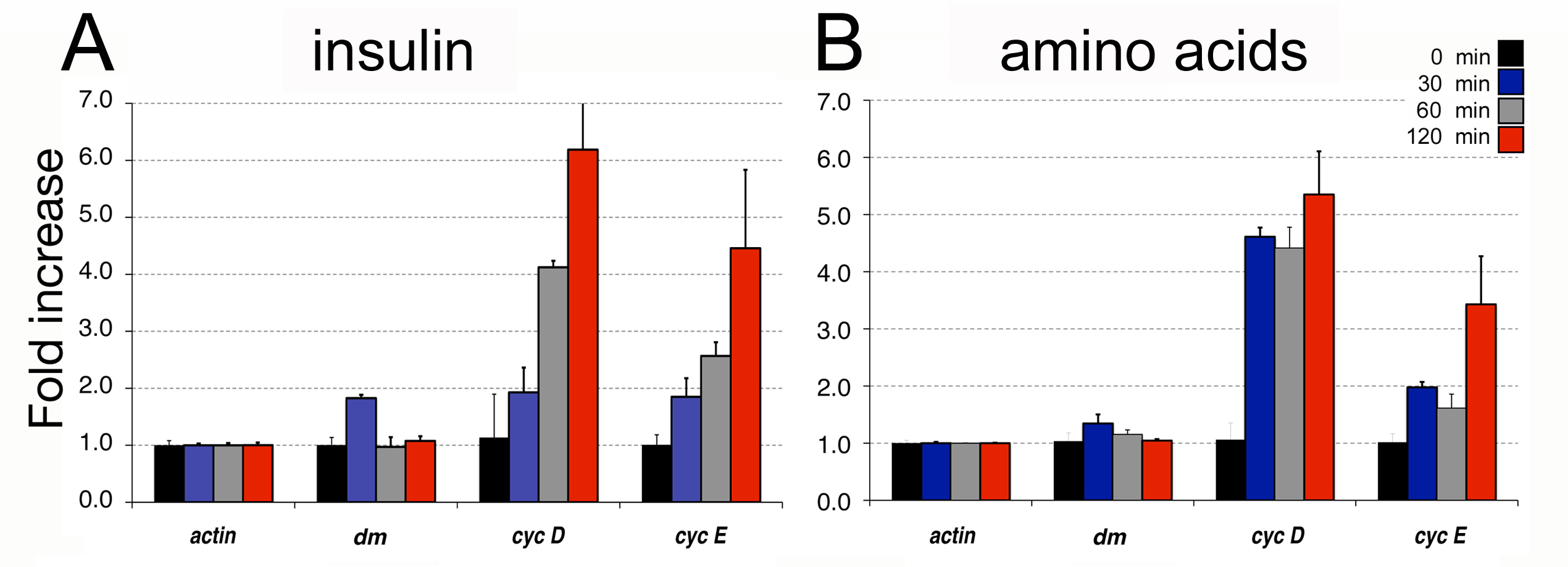

Supplement: Additional file 1 — Quantitative RT-PCR comparing the transcript levels for dm, cyclin D and cyclin E in Drosophila S2 cells upon insulin (A) or amino acids (B) treatment. Cells were treated with insulin or AAs and RNA was extracted at the indicated times. qRT-PCRs were performed to analyze expression of diminutive (dm), cyclin D and cyclin E RNAs. The sequences of the primers used are available in Additional file 8; Supplementary Material and Methods. actin was used as the internal control. Error bars indicate the standard deviations (±) calculated on the average of three separate experiments. [file 1741-7007-9-65-S1.JPEG]

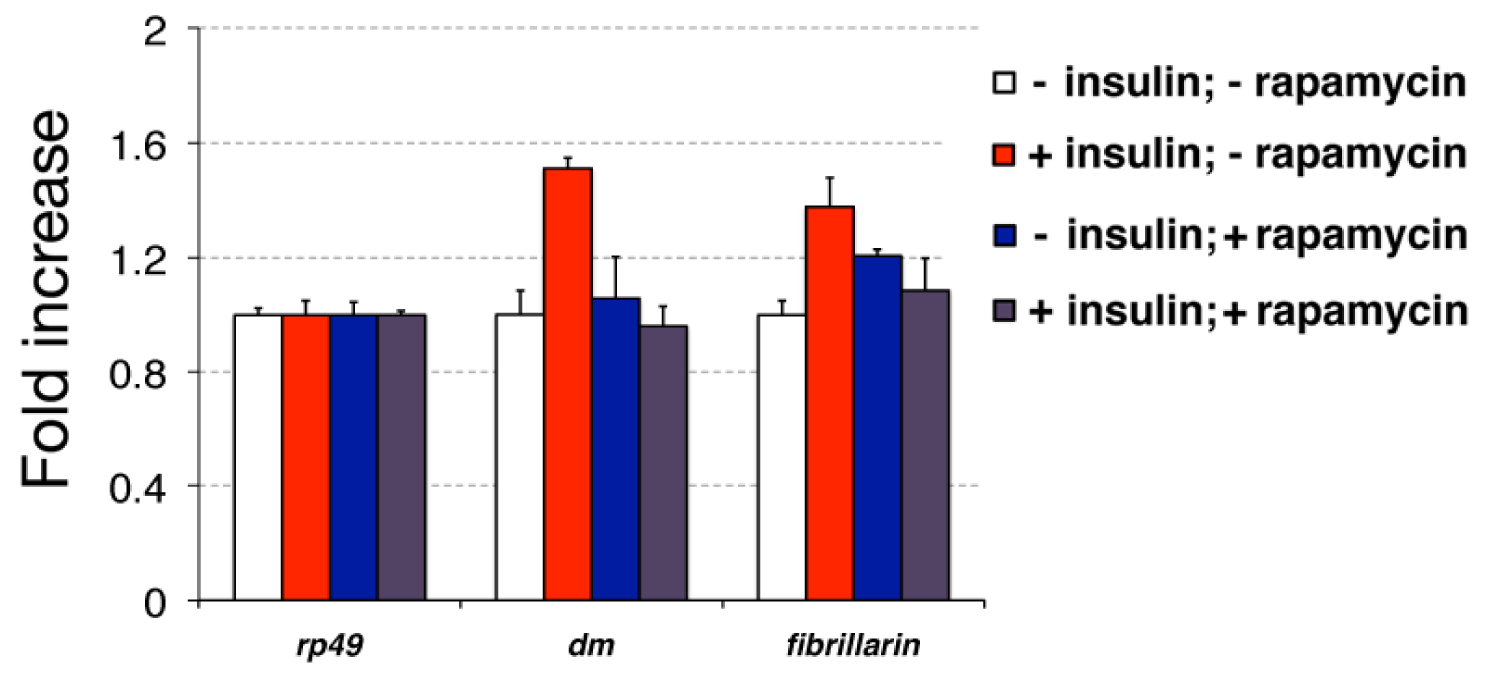

Supplement: Additional file 2 — Quantitative RT-PCR of dm and its target fibrillarin in Drosophila S2 cells upon insulin treatments and in the presence of rapamycin. Cells were treated with insulin or rapamycin alone and together as indicated in the figure; rp49 (ribosomal protein 49) was used as internal control. Similar results were obtained using actin as a control (not shown). Error bars indicate the standard deviation (±) calculated from three independent experiments. [file 1741-7007-9-65-S2.JPEG]

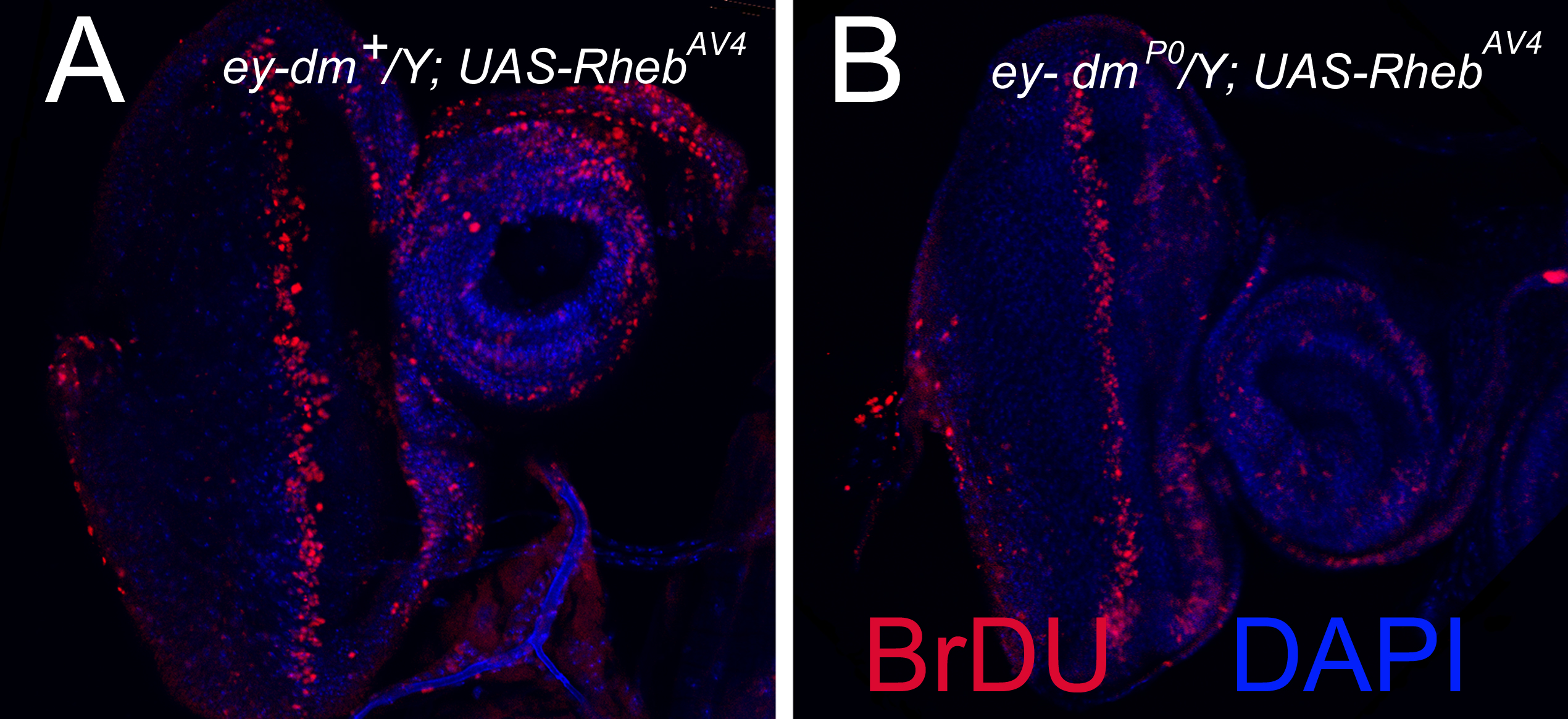

Supplement: Additional file 6 — BrdU-labeling in the eye imaginal discs from third instar larvae expressing RhebAV4 transgene in wild-type dm+/Y (A) or in hypomorphic dmP0/Y animals (B). Expression of the UAS-RhebAV4 did not significantly alter the S phase in the cells of the eye imaginal disc, visualized by BrdU labeling (red). Nuclei are labeled with DAPI (blue). Posterior is to the left. [file 1741-7007-9-65-S5.JPEG]

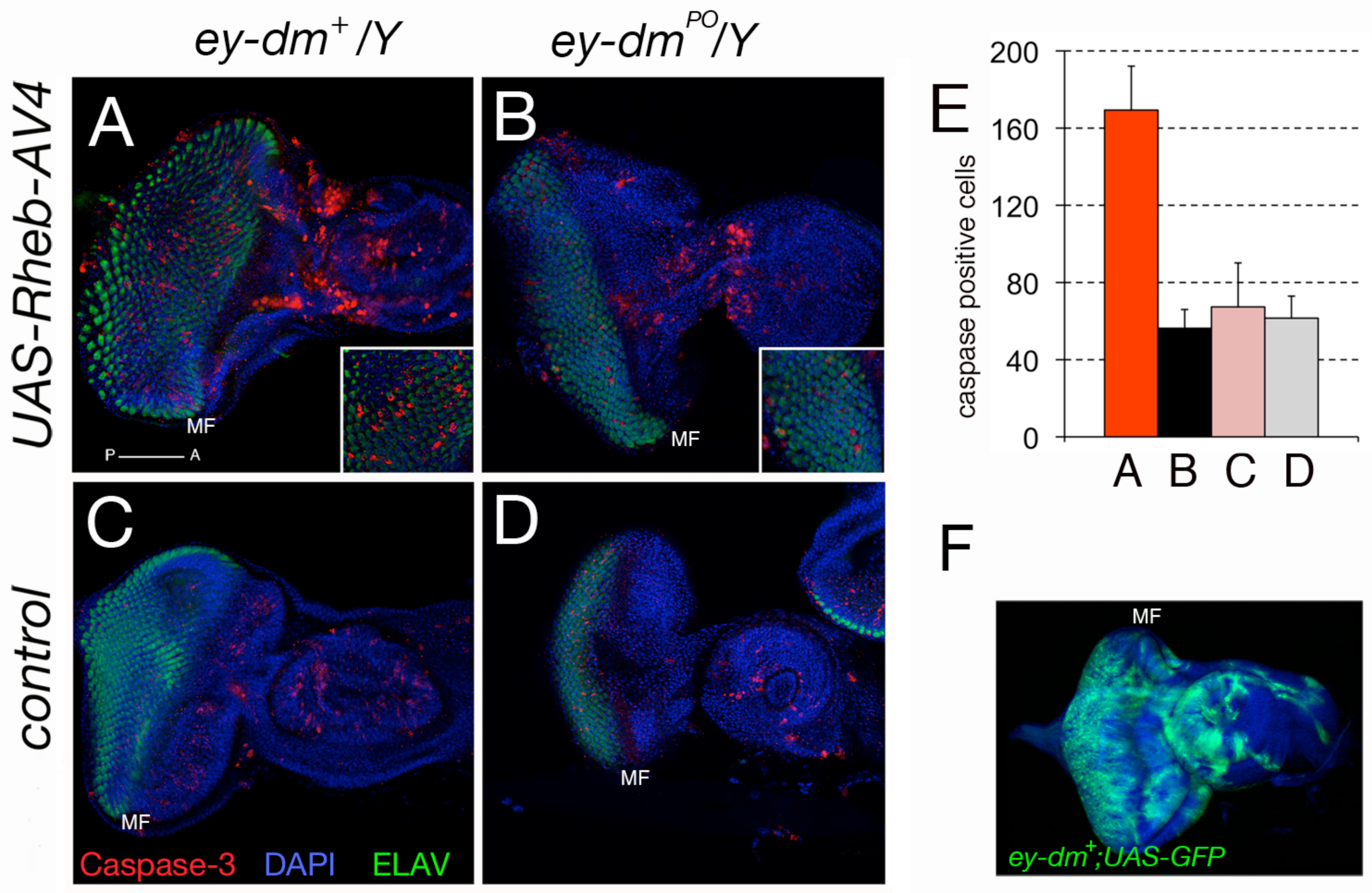

Supplement: Additional file 7 — Expression of RhebAV4 induces Myc-dependent apoptosis. Third instar eye imaginal discs from ey-dm+/Y or ey-dmP0/Y larvae carrying the UAS-RhebAV4 transgene (A-B) or control chromosome (C-D) were tested for the presence of apoptotic cells. Discs were stained with anti-active caspase 3 antibody (red) to visualize cell death, or with anti ELAV (green) to mark the differentiated neuronal cells posterior to the morphogenetic furrow (MF). DAPI staining (blue) indicates nuclei. (E) Quantification of caspase-positive cells in the region posterior to the MF of the indicated genotype (visible in insets). Error bars indicate standard deviation (±) calculated from six independent eye imaginal discs. P < 0.001for t test for ey- dm+/Y; UAS-RhebAV4 vs. ey- dmP0/Y; UAS-RhebAV4 while comparisons within the other genotypes resulted in P > 0.1. (F) Photo of an eye imaginal disc from third-instar ey- dm+/Y; UAS-GFP larvae highlighting the territory where the eyeless-Gal4; UAS-GFP transgene is expressed. Posterior is to the left. [file 1741-7007-9-65-S6.JPEG]

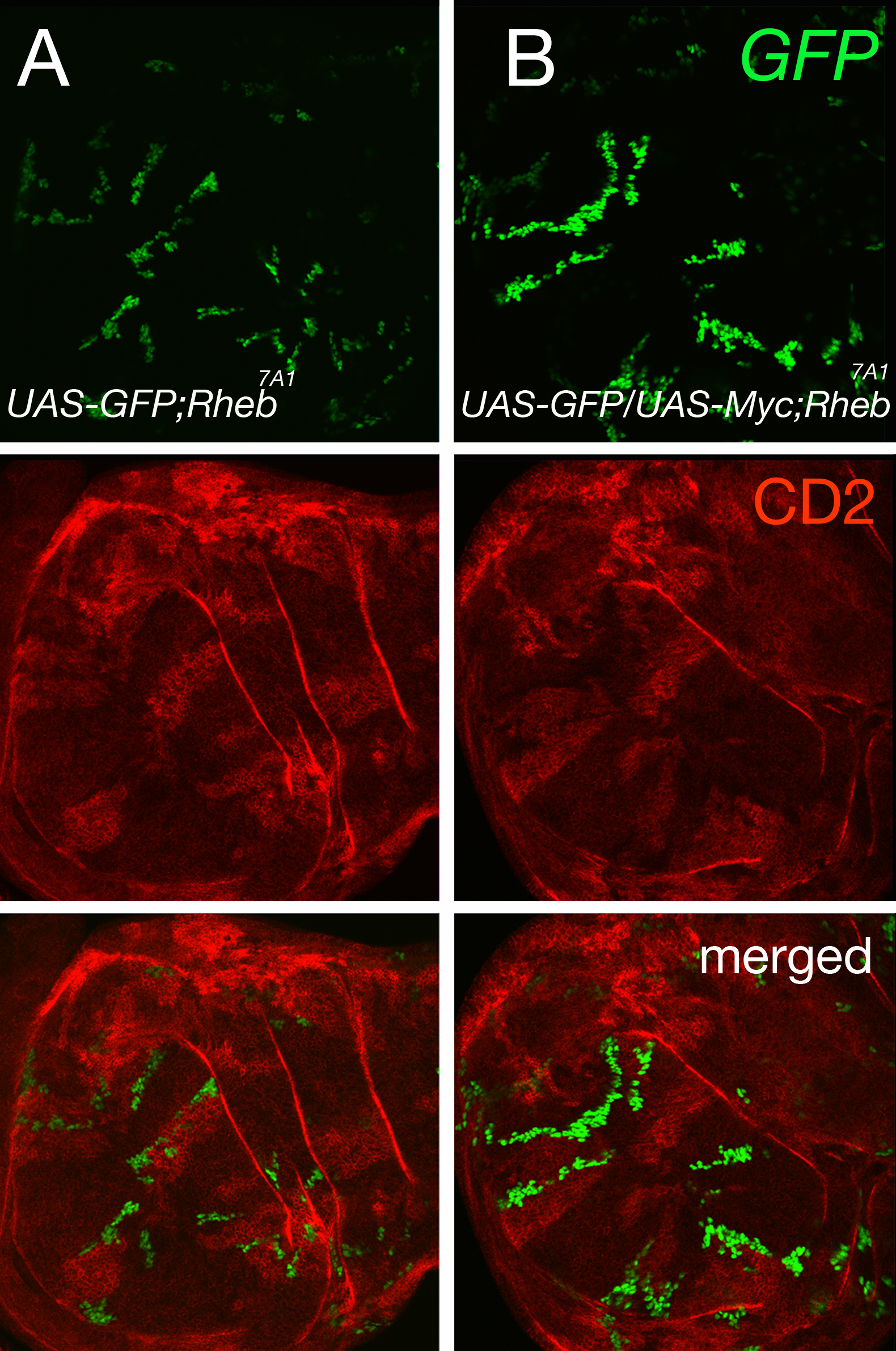

Supplement: Additional file 8 — MARCM showing a partial rescue of Rheb7A1 hypomorphic mutant clones by Myc overexpression. Rheb7A1 homozygous mutant clones suffer of growth disadvantage [39]. These clones, induced at 72 h AEL and marked by GFP expression, are significantly smaller than wild-type siblings, which are marked by CD2 staining (red). The growth defect of Rheb7A1 mutant clones (A) is partially rescued by expression of Myc (B). Those clones are visible by co-expression with GFP, while wild-type clones are marked by the expression of CD2 and visualized by immunofluorescence using anti-CD2 antibodies. To generate MARCM clones the line hs-flp, tub-Gal4 (w+), UAS-GFP (w+); FRT82 [hsCD2 (y+)] tub-Gal80 was crossed with the line w; FRT82 Rheb7E1/TM6b (A) or with w; UAS-dMyc; FRT82 Rheb7E1/TM6b (B). (see Supplementary Material and Methods in Additional file 9). [file 1741-7007-9-65-S7.JPEG]
