# Supplementary material for: Drosophila insulin and target of rapamycin (TOR) pathways regulate GSK3 beta activity to control Myc stability and determine Myc expression in vivo
Source: BMC Biol. 2011 Sep 27;9:65. doi: 10.1186/1741-7007-9-65 (PMC3235970; doi:10.1186/1741-7007-9-65)
Supplement: Additional file 3 — Appendix 1. Significance analysis of the conjectured growth for ommatidial number and size used in the experiments outlined in Figure 4 and Table 1. The P-values of a two-tailed z test for the analysis are represented in Table S1 in Additional file 4. [file 1741-7007-9-65-S3.PDF]

Appendix 1: A significance analysis

1 The problem

The over-expression of n_g genes $j = 1, \ldots, n_g$ leads to both growth and proliferation of ommatidia in the eyes of fruit flies. The goal of this study is to understand whether and how this growth depends on the alteration of n_b other background genes $i = 1, \ldots, n_b$. We shall denote by $j = 0$ and $i = 0$ the wild cases where no gene has been altered. For each (i, j), we have n_{ij} observations of the cell's size S_{ij} and number N_{ij}:

$$S_{ij}^k, N_{ij}^k, \ k = 1, \ldots, n_{ij}.$$

The relative rates of growth α_{ij} and proliferation β_{ij} are then defined by

$$\alpha_{ij} = \frac{S_{ij}}{S_{i0}}$$

and

$$\beta_{ij} = \frac{N_{ij}}{N_{i0}}.$$

Our goal is to estimate these rates from the observations, and to make a significance analysis to determine whether one can conclude from the data that α_{ij} and β_{ij} depend on i, the background genetic alteration.

To this end, we need to figure out the level of uncertainty in our estimation of the α's and β's. Since the mathematics is the same for both, and also independent of which mutation (i, j) we are looking at, we shall formulate the following generic problem:

Problem (preliminary version): Given n measurements $x_j, \ j = 1, \ldots, n$ of a variable x (the wild population), m measurements $y_i, \ i = 1, \ldots, m$ of a variable y (one gene over-expressed), and a conjectured growth model

$$y = \alpha x, \tag{1}$$

estimate α and its level of uncertainty $\Delta\alpha$ (The coefficient α here represents either α_{ij} or β_{ij} above.)

Once these values are known for two different original alterations, verifying that the corresponding α's are different within a given degree of confidence can be done with a simple test of hypothesis.

The subtle part of this problem is that we do not have joint observations (x_i, y_i), since each animal is or not genetically altered ad initio. Instead, we measure x_j for some animals, and y_i for some others, whose corresponding values of x are unknown. We will denote these unknown values by z_i, corresponding to the hypothetical measurements of wild animals that, under the alteration studied, have sizes y_i.

It is not enough to propose the model (**??**) to solve the problem; some hypothesis on the errors involved is also required. Since we should expect larger variations for larger animals, we propose

$$y = (\alpha + \epsilon)x. \tag{2}$$

where the individual variation ϵ is a statistical variable independent of x, with zero mean and standard deviation σ_ϵ. Then $\langle \epsilon x \rangle = 0$, and

$$\alpha = \frac{\langle y \rangle}{\langle x \rangle}.$$

Let us now formulate the mathematical problem more precisely:

Problem (final version): Given independent measurements $x_j,\ (j = 1, \ldots, n)$ and $y_i,\ (i = 1, \ldots, m)$ of two variables x and y, and a conjectured growth model (**??**), find estimates $\hat{\alpha}$ and $\hat{\sigma_\epsilon}$ for α and σ_ϵ, and the standard deviation $\Delta\alpha$ of the estimate $\hat{\alpha}$.

2 The estimations

We are given independent observations of x and y, and we introduce the unobservable –or hidden– variable z and the variability ϵ, so that, for each observation i of y,

$$y_i = (\alpha + \epsilon_i)z_i. \tag{3}$$

2.1 $\hat{\alpha}$

A naive estimate for α would be given by

$$\alpha_{est} = \frac{\bar{y}}{\bar{x}} = \frac{\frac{1}{m}\sum_{i=1}^{m} y_j}{\frac{1}{n}\sum_{j=1}^{n} x_i}. \tag{4}$$

It turns out, however, that this estimate is biased. To see this, compute the expected value of α_{est} under the model (**??**), denoting by μ_x and σ_x the expected value and standard deviation of x (and also of z, which represents the same variable in situations where it cannot be observed):

$$\langle \alpha_{est} \rangle \quad = \quad \left\langle \frac{\frac{1}{m}\sum_{i=1}^{m}(\alpha + \epsilon_i)z_i}{\frac{1}{n}\sum_{j=1}^{n} x_j} \right\rangle = \left\langle \frac{\frac{1}{m}\sum_{i=1}^{m}(\alpha + \epsilon_i)\,(\mu_x + \delta z_i)}{\frac{1}{n}\sum_{j=1}^{n}\,(\mu_x + \delta x_j)} \right\rangle$$

$$= \left\langle \frac{1}{1+\frac{1}{n}\sum_{j=1}^{n}\frac{\delta x_j}{\mu_x}} \right\rangle \alpha \approx \left(1+\frac{1}{n}\left(\frac{\sigma_x}{\mu_x}\right)^2\right)\alpha.$$

It follows that a better estimate for α is given by

$$\hat{\alpha} = \frac{1}{\left(1+\frac{1}{n}\left(\frac{\Delta x}{\bar{x}}\right)^2\right)} \frac{\bar{y}}{\bar{x}}, \tag{5}$$

where

$$\Delta x = \sqrt{\frac{1}{n-1}\sum_{j=1}^{n}(x_j-\bar{x})^2} \tag{6}$$

is an unbiased estimate for σ_x.

2.2 $\hat{\sigma_\epsilon}$

Estimating σ_ϵ, the standard deviation of the variability of the relative rate of growth α, is quite straightforward. It follows from (**??**) that

$$\langle y \rangle = \alpha \langle x \rangle,$$

so

$$\sigma_y^2 = \left\langle (y-\langle y\rangle)^2 \right\rangle = \left\langle (\alpha(x-\langle x\rangle)+\epsilon x)^2 \right\rangle = \alpha^2\sigma_x^2 + \sigma_\epsilon^2\left(\mu_x^2+\sigma_x^2\right).$$

Hence we can propose the estimate

$$\hat{\sigma_\epsilon} = \sqrt{\max\left(\frac{(\Delta y)^2 - \hat{\alpha}^2(\Delta x)^2}{\bar{x}^2+(\Delta x)^2}, 0\right)},$$

where

$$\Delta y = \sqrt{\frac{1}{m-1}\sum_{i=1}^{m}(y_i-\bar{y})^2}. \tag{7}$$

2.3 $\Delta\alpha$

Next we compute the uncertainty $\Delta\alpha$ in our estimation of α:

$$\begin{aligned}
(\Delta\alpha)^2 &= \left\langle (\hat{\alpha}-\alpha)^2 \right\rangle = \left\langle \left(\frac{1}{1+\frac{1}{n}\left(\frac{\Delta x}{\bar{x}}\right)^2} \frac{\frac{1}{m}\sum_{i=1}^{m}(\alpha+\epsilon_j)z_j}{\frac{1}{n}\sum_{j=1}^{n}x_i} \right)^2 \right\rangle - \alpha^2 \\
&= \left(\left\langle \left(\frac{1}{1+\frac{1}{n}\left(\frac{\Delta x}{\bar{x}}\right)^2}\right)^2 \left(\frac{1+\frac{1}{m}\sum_{i=1}^{m}(1+\frac{\epsilon_j}{\alpha})\left(\frac{z_j}{\mu_x}-1\right)}{1+\frac{1}{n}\sum_{j=1}^{n}\left(\frac{x_i}{\mu_x}-1\right)} \right)^2 \right\rangle - 1 \right)\alpha^2 \\
&\approx \left(\left(1-\frac{2}{n}\left(\frac{\sigma_x}{\mu_x}\right)^2\right) \left\langle \left(\frac{1+\frac{1}{m}\sum_{i=1}^{m}(1+\frac{\epsilon_j}{\alpha})\left(\frac{z_j}{\mu_x}-1\right)}{1+\frac{1}{n}\sum_{j=1}^{n}\left(\frac{x_i}{\mu_x}-1\right)} \right)^2 \right\rangle - 1 \right)\alpha^2
\end{aligned}$$

For the term within the brackets, we have

$$\langle \ldots \rangle \approx 1 + \left[\frac{1}{m} \left(1 + \left(\frac{\sigma_\epsilon}{\alpha} \right)^2 \right) + \frac{3}{n} \right] \left(\frac{\sigma_x}{\mu_x} \right)^2 .$$

It follows that

$$\frac{\Delta\alpha}{\alpha} \approx \sqrt{\frac{1}{n} + \frac{1}{m} \left(1 + \left(\frac{\sigma_\epsilon}{\alpha} \right)^2 \right)} \, \frac{\sigma_x}{\mu_x}, \tag{8}$$

which can be estimated replacing α, μ_x, σ_x and σ_ϵ by their estimates $\hat{\alpha}$, $\bar{x}$, Δx and $\hat{\sigma_\epsilon}$ above.

3 Analysis of significance

Now we are ready to test with what level of confidence two values of α, corresponding to two different background gene perturbations, can be concluded to be different based on the available observations. Carrying out the estimations above, we have values for $\hat{\alpha}_{1,2}$ and $\Delta\alpha_{1,2}$. Using a standard two-sample z-test, we define

$$z = \frac{\hat{\alpha}_1 - \hat{\alpha}_2}{\sqrt{(\Delta\alpha_1)^2 + (\Delta\alpha_2)^2}} \tag{9}$$

and compute the two-tailed p-value from

$$p = 2\Phi(-|z|), \tag{10}$$

where Φ is the normal cumulative distribution function. The smaller p, the more likely that the two values of α are different; typical cut-off values for p are one and five percent.
