# Supplementary material for: Drosophila insulin and target of rapamycin (TOR) pathways regulate GSK3 beta activity to control Myc stability and determine Myc expression in vivo
Source: BMC Biol. 2011 Sep 27;9:65. doi: 10.1186/1741-7007-9-65 (PMC3235970; doi:10.1186/1741-7007-9-65)
Supplement: Additional file 4 — Table S1. Representation of the relative increase of the size for each ommatidium and their total number. The values represent the percentage of increase in size (a-Size) or number (a-Number) as compared to their control and relative genetic background (see also data in Table 1). da is the standard deviations and the total number of animals used is indicated in Table 1. To establish the significance of the relative differences of the data within the different dm genetic background, we calculate the P-values using a standard two-sided z-test (the formula used is represented in Appendix 1 of Additional file 3). * complete genotype: the construct tubulin-FRT-dmyc-cDNA-FRT-Gal4, ey-Flp/Y was recombined into the dm+, dmpoor dm4 genetic background. [file 1741-7007-9-65-S4.DOC]

| **Relative Size of each Ommatidium Size** | | | | | |
| --- | --- | --- | --- | --- | --- |
| **Genotypes *** | ***Dp110*** | ***PTEN*** | ***S6K*** | ***Rheb- ^Av4^*** | ***TOR- ^TED^*** |
| **a-Size** | | | | | |
| *1 ey- dm*^+^ */ Y; +/+* | 138 | 092 | 105 | 189 | 089 |
| *2 ey- dm*^P0^*/ Y; +/+* | 135 | 064 | 099 | 154 | - |
| *3 ey- dm*^4^*/ Y; +/+* | 127 | 067 | - | 134 | - |
| **da** | | | | | |
| *1 ey- dm*^+^ */ Y; +/+* | 3.00 | 1.77 | 1.90 | 4.17 | 1.26 |
| *2 ey- dm*^P0^*/ Y; +/+* | 2.82 | 1.35 | 1.70 | 2.87 | - |
| *3 ey- dm*^4^*/ Y; +/+* | 4.20 | 2.22 | - | 4.03 | - |
| ***p-value*s** | | | | | |
| *1 versus 2* | 0.4292 | 0.0000 | 0.0228 | 0.0000 | - |
| *1 versus 3* | 0.0367 | 0.0000 | - | 0.0000 | - |
| *2 versus 3* | 0.1355 | 0.0317 | - | 0.0000 | - |
| **Relative Increase of the Ommatidia Number** | | | | | |
| **Genotypes *** | ***Dp110*** | ***PTEN*** | ***S6K*** | ***Rheb- ^Av4^*** | ***TOR- ^TED^*** |
| **a-Number** | | | | | |
| *1 ey- dm*^+^ */ Y; +/+* | 112 | 074 | 095 | 076 | 080 |
| *2 ey- dm*^P0^*/ Y; +/+* | 096 | 061 | 093 | 088 | - |
| *3 ey- dm*^4^*/ Y; +/+* | 099 | 070 | - | 100 | - |
| **da** | | | | | |
| *1 ey- dm*^+^ */ Y; +/+* | 1.12 | 0.65 | 0.90 | 1.03 | 0.73 |
| *2 ey- dm*^P0^*/ Y; +/+* | 1.10 | 0.67 | 0.90 | 0.86 | - |
| *3 ey- dm*^4^*/ Y; +/+* | 2.58 | 0.28 | - | - | - |
| ***p-value*s** | | | | | |
| *1 versus 2* | 0.0000 | 0.0000 | 0.0642 | 0.0000 | - |
| *1 versus 3* | 0.0000 | 0.1215 | - | 0.0000 | - |
| *2 versus 3* | 0.2797 | 0.0001 | - | 0.0000 | - |
