# Supplementary material for: Drosophila insulin and target of rapamycin (TOR) pathways regulate GSK3 beta activity to control Myc stability and determine Myc expression in vivo
Source: BMC Biol. 2011 Sep 27;9:65. doi: 10.1186/1741-7007-9-65 (PMC3235970; doi:10.1186/1741-7007-9-65)
Supplement: Additional file 5 — Table S2. Analysis of the ommatidial size and number in animals with different dm genetic background. The total number of ommatidia and the relative size of each ommatidium are indicated. Standard deviations (±) are calculated based on the total number of the animals reported in parenthesis. Values represent the relative increase in the size (a) or number (b) of the ommatidia compared to the values in their genetic background (100). P-values are calculated from Student t test and are reported for the calculation of ommatidia number (c) and size (d). * complete genotype: the construct tubulin-FRT-dmyc-cDNA-FRT-Gal4, ey-Flp/Y was recombined into the dm+, dmpoor dm4 genetic background. [file 1741-7007-9-65-S9.DOC]

| **Genotype** | | **total number** | **size (µm^2^)** | **%**  **number (a)** | **%**  **size**  **(b)** | ***p-*values**  **number**  **(c)** | ***p-*values**  **size**  **(d)** |
| --- | --- | --- | --- | --- | --- | --- | --- |
| *1* | *ey *- dm*^+^ */ Y* | 726 ± 17 (15) | 239 ± 11 (15) | 100 | 100 | - | - |
| *2* | *ey *- dm*^P0^*/ Y* | 735 ± 16 (12) | 218 ± 9 (12) | 101 | 092 | (1*vs* 2) 0.14530 | (1*vs* 2) 0.00003 |
| *3* | *ey *- dm*^4^ */ Y* | 650 ± 44 (17) | 179 ± 11 (10) | 089 | 078 | (1*vs* 3) 0.00000 | (1*vs* 3) 0.00000 |
