# Supplementary material for: Drosophila insulin and target of rapamycin (TOR) pathways regulate GSK3 beta activity to control Myc stability and determine Myc expression in vivo
Source: BMC Biol. 2011 Sep 27;9:65. doi: 10.1186/1741-7007-9-65 (PMC3235970; doi:10.1186/1741-7007-9-65)
Supplement: Additional file 9 — Supplementary Material and Methods. [file 1741-7007-9-65-S8.DOC]

**Supplementary Material and Methods**

**Quantitative real-time PCR**

S2 cells were collected under the indicated conditions and total RNA was extracted using the RNeasy kit (Qiagen) and on-column DNase digested to eliminate genomic DNA contamination. Reverse transcription was carried out with random primers using 1 µg of RNA per sample using the Superscript II Reverse Transcriptase (Invitrogen). For quantitative real-time PCR, we used Quantitect SYBR green PCR kit (Qiagen) in an ABI 7900HT thermal cycler. Reactions were carried out using the following primer pairs: *actin:* 5’-AAACGCGGTTCTGCATGAG-3’(R), 5’-GACGCTTCAAGGGACAGTATCTG-3’(F); *dmyc*: 5’ CATAACGTCGACTTGCGTG-3’ (R), 5’-GAAGCTCCCTGCTGATTTGC-3’ (F); *fibrillarin:* 5’- ATGCGGTACTTGTGTGGATG -3’(R), 5’- ACGACAGTCTCGCATGTGTC -3’(F); *cycE:* 5’-TTTGGCCTGGGACTAATCTG-3’(R), 5’-CGTCAGAAGACTTTCGGGAG-3’(F); *cycD*: 5’-GGATCCCACATTGTATTCGG-3’(R), 5’- ACGGAGCTTTGAAGCCAGTA -3’(F). All PCR reactions were carried out in triplicate and control PCR reactions were performed using templates that had been mock reverse-transcribed (no reverse transcriptase). The control reactions confirmed that genomic DNA had been efficiently removed.

**Immunofluorescence and BrdU staining**

Third-instar larvae were dissected in 1X PBS. Eye or wing discs were collected and fixed in 4% paraformaldehyde/PBS and permeabilzed in 0.3% Triton X-100 prior to incubation with specific antibodies. Primary antibodies were used at a dilution of 1:5 for mouse anti-dMyc, 1:1000 for anti-CD2 (Myriam Zecca, Columbia University), 1:250 for rabbit anti-active caspase-3 (Cell Signaling), 1:1000 for rat anti-ELAV or anti-BrdU (DHSB). Primary antibodies incubations were performed at 4°C in 3% BSA/PBT. Secondary antibodies used: anti-mouse Alexa-555, anti-rat Alexa-488 and anti-rabbit Alexa-555 (Invitrogen). Imaging analysis was performed with a Zeiss LSM 510 Confocal Microscope. For BrdU analysis eye discs were incubated for 30 minutes in a solution containing 200 mg/ml of BrdU in PBS before fixation. Discs were then processed as described above.

**Quantification of caspase-3 positive cells in the eye imaginal discs**

Cells positive for caspase-3 staining were calculated from photos from six individual eye imaginal discs from each of the genotypes. Total number of positive cells was calculated from Photoshop photos-images using fix grid and only in the area posterior to the morphogenetic furrow, where single cells could be identified. Statistics was applied and standard deviation was calculated from six animals.

**Generation of MARCM clones**

For MARCM analysis, the repressor Gal80 was expressed under the constitutive *tubulin* promoter allowing functional repression of Gal4 transcriptional activator. At 72 hours after egg laying (AEL), larvae were heat-shocked to induce the event of recombination, which resulted in the formation of *Rheb^7A1^* mutant clones (and their sibling *wild-type* twin-spots). The event of recombination also resulted in the elimination of tub>Gal80 in *Rheb^7A1^* mutant clones, thus allowing the release of Gal4 and induction of *UAS-Myc* expression in the mutant tissue. *Wild-type* twin-spot clones were visualized using anti-CD2 antibodies, *UAS-Myc* expressing cells or *Rheb^7A1^* mutant clones were marked by the expression of *tubulin>Gal4 UAS-GFP*. To generate MARCM clones the line *w;* *hs-Flp; tub-Gal4, UAS-GFP; FRT82 (hs-CD2 y+)-tub-Gal80 (w+)* was crossed with the line *w; FRT82 Rheb^7E1^/TM6b* **(A)** or with *w; UAS-dMyc; FRT82 Rheb^7E1^/TM6b* **(B).**
